# GMP compliant isolation of mucosal epithelial cells and fibroblasts from biopsy samples for clinical tissue engineering

**DOI:** 10.1038/s41598-021-91939-0

**Published:** 2021-06-11

**Authors:** Angela Tait, Toby Proctor, Nick J. I. Hamilton, Martin A. Birchall, Mark W. Lowdell

**Affiliations:** 1 Cancer Institute, Department of Haematology, University College London, London, UK; 2 Department of Biochemical Engineering, University College London, London, UK; 3 Institute of Ophthalmology, University College London, London, UK; 4 UCL Ear Institute, University College London, London, UK

**Keywords:** Skin stem cells, Stem-cell niche, Skin models, Fluorescence imaging, Multicellular systems, Cell culture, Flow cytometry, Histology, Tissue culture, Molecular medicine, Confocal microscopy

## Abstract

Engineered epithelial cell sheets for clinical replacement of non-functional upper aerodigestive tract mucosa are regulated as medicinal products and should be manufactured to the standards of good manufacturing practice (GMP). The current gold standard for growth of epithelial cells for research utilises growth arrested murine 3T3 J2 feeder layers, which are not available for use as a GMP compliant raw material. Using porcine mucosal tissue, we demonstrate a new method for obtaining and growing non-keratinised squamous epithelial cells and fibroblast cells from a single biopsy, replacing the 3T3 J2 with a growth arrested primary fibroblast feeder layer and using pooled Human Platelet lysate (HPL) as the media serum supplement to replace foetal bovine serum (FBS). The initial isolation of the cells was semi-automated using an Octodissociator and the resultant cell suspension cryopreservation for future use. When compared to the gold standard of 3T3 J2 and FBS containing medium there was no reduction in growth, viability, stem cell population or ability to differentiate to mature epithelial cells. Furthermore, this method was replicated with Human buccal tissue, providing cells of sufficient quality and number to create a tissue engineered sheet.

## Introduction

Airway reconstruction for patients with severe stenosis (narrowing) or malacia (lack of rigidity) often involves replacing damaged upper aerodigestive tract mucosa, with split skin grafts^1^. Although convenient and effective, split skin cannot replicate normal mucosa. Such grafts lead to mucus stasis, infection, halitosis and coughing, and can even generate hairs with further morbidity^2,3^. Harvesting a split skin graft creates weeks of severe pain and long-term disfigurement in the donor site (commonly thigh or abdominal wall)^4^. Thus, a tissue-engineered, non-keratinised epithelial sheet might offer an attractive alternative and would better recapitulate the internal luminal environment.

The production of epithelial cell sheets for clinical use needs to be carried out under the regulatory and quality umbrella of good manufacturing practice (GMP). This is the minimum standard that a medicines manufacturer must meet in their production process, with specific regulations for human cell-based medicinal products^5^. The regulations state that the use of animal reagents should be avoided and replaced by non-animal derived reagents, such as irradiated sera or synthetic media and that raw materials should be of pharmaceutical grade where possible^6^. Human pooled platelet lysate (HPL) is a rich source of cytokines, chemokines and growth factors^7^ and has been shown to substitute for feeder effects, supporting MSC growth while maintaining phenotype and function^8–10^. Various epithelial cell lines and gingival fibroblasts proliferate in 5% HPL^11–13^. It has been used clinically for provision of MSC to treat graft-versus-host-disease^14,15^ and is available as a GMP compliant product^16^.

Present in vitro epithelial models rely on the use of a murine fibroblast feeder layer (3T3 J2)^17^ and/or a culture medium containing animal products such as foetal bovine serum (FBS)^18^, bovine pituitary extract (BPE) and cholera toxin^19^. There is a commercially available epithelial advanced therapy medicinal product (ATMP)^20^ created using a 3T3-J2 feeder layer from a GMP-certified master cell bank^21^, which is exclusive to the company and would be very expensive to create independently^22^. Although non-GMP compliant 3T3 J2 feeders have been allowed in special clinical cases^23^, However cells grown on a 3T3 feeder layer in FBS containing medium have been shown to contain a non-human form of sialic acid (Neu5Gc) which elicits adverse immune responses in humans^24^. Thus the concerns arising from the use of xenogeneic material in patients has led to research into alternatives for the growth of squamous epithelial cells, such as growth arrested dermal fibroblasts^25^, MSC’s^26^ or omitting feeder layers all together^27^.

Buccal mucosa has been used as a source of non-keratinised squamous epithelial cells, either as a graft^28,29^ or as a tissue engineered product^30^. Various methods for procurement and culture of mucosal cells, such as outgrowth^31^ or enzymatic digestion^18^, use of feeder layers^32^ or tissue culture plastic coatings^19^ and various media^33^ have been reported. Green and Rheinwald initially cultured keratinocytes on growth-arrested 3T3 J2^17^, which have been shown to also promote the growth of oral epithelial cells^34^.

The objective of this study was to engineer a GMP-compliant process for the isolation and expansion of primary, non-keratinised squamous epithelial cells from buccal mucosa for potential clinical use. Here, we used porcine aerodigestive mucosa for proof-of-principle work. The pig is an ideal large animal to model upper aerodigestive research questions, their mucosa has comparable morphological characteristics to human aerodigestive mucosa^35^. Once an appropriate method had been achieved it was repeated with human buccal biopsies.

## Results

### Fibroblasts

Removal of the epithelial sheet from the lamina propria was investigated on both supraglottal and buccal porcine mucosa. After incubation in neutral protease for 16 ± 3 h at 4 °C followed by 10 min at room temperature the sheets were easily removed.

**Figure 1:**
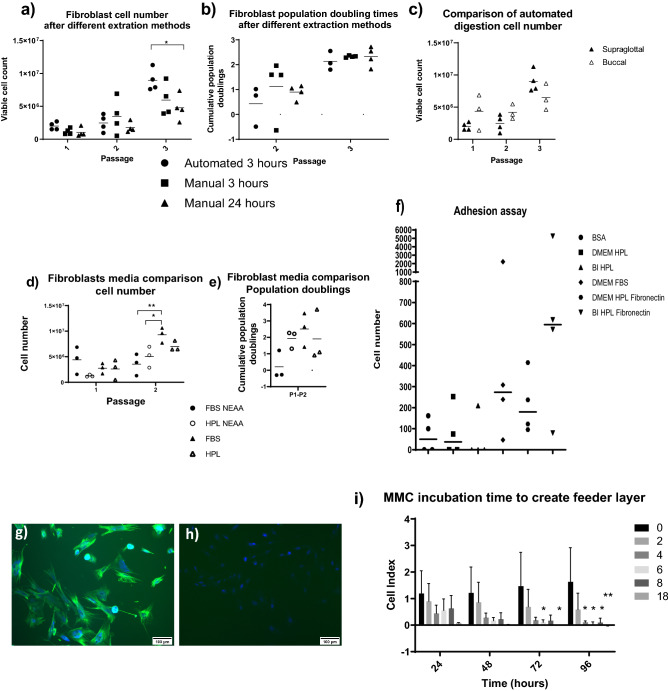
Fibroblasts. Supraglottal fibroblasts were extracted using 3 different methods, the viable cell number (**a**) and the population doublings (**b**) were recorded up to passage 3. Data are mean plus data points n = 4. *P ≤ 0.05 (Two way ANOVA with Tukey’s multiple comparisons). Buccal tissue was extracted via automated method and compared to supraglottal tissue (**c**), there was no significant difference between tissue type. Following extraction buccal fibroblasts were grown in 4 compositions of media, DMEM containing either FBS NEAA, HPL NEAA, FBS or HPL, cell number (**d**) and population doubling (**e**) were recorded up to passage 2. FBS medium had a significantly higher cell number than both NEAA containing medium by passage 2. Data are mean plus data points, n = 3 *P < 0.05 **P < 0.01 (two way ANOVA with Tukey’s multiple comparison). Adhesion of buccal fibroblasts was examined (**f**). Cells were fluorescently labelled with Calcein AM and added to a 96 well plate with BSA coating as a negative control, fibroblast medium containing HPL and FBS compared to Bi HPL medium, and fibronectin coating with both HPL containing media for 3 h. After washing, the plate was read. Data are median plus data points *n* = 4. Fibrolast were stained with fibroblast marker vimentin (**g**) and myofibroblast marker αSMA (**h**) n = 3, scale bar shows 100 μm. MMC incubation time for the creation of feeder layers (**i**) Cells were treated with MMC for 0, 2, 4, 6, 8 and 18 h before being placed into the xCELLigence plate reader. Readings were taken every hour for 96 h. Data are mean ± SD, *n* = 3. *P < 0.05 **P < 0.01, for comparison to untreated control (two way ANOVA and Tukey’s multiple comparison).

Digestion of the lamina propria to produce fibroblasts requires 24 h in collagenase at 37 °C or using the “h_skin_1” protocol for the Octodissociator a 3 h 37 °C enzymatic digestion before dissociation. The cells were then seeded into FBS-non-essential amino acid (NEAA) medium. The incubation time and methods were initially investigated using porcine supraglottal tissue, the viable cell number obtained was slightly greater for the automated method, providing significantly more cells by passage three (Fig. 1A), however there was no significant difference in cumulative population doublings between the three methods tested (Fig. 1B), suggesting that there were more cells obtained. The automated method was used on porcine buccal tissue and compared to the supraglottal, there was no significant difference between the two tissue types in the number of cells achieved up to passage 3 (Fig. 1C). All further experiments used the automated method and buccal tissue.

The composition of fibroblast media was investigated using buccal fibroblasts. Media containing FBS with NEAA, HPL with NEAA, FBS and HPL only were compared, by passage 2 there were significantly greater cells in the FBS medium compared to those containing NEAA. There was no significant difference between FBS and HPL. However there was no significant difference in population doublings achieved suggesting that a lower cell number was obtained at p0 due to varying adhesion (Fig. 1D,E).

When obtaining fibroblasts from human buccal pinch biopsies, there was no cell growth although cells were present in the digested material, therefore adhesion was investigated in porcine buccal fibroblasts.

FBS increased adhesion compared to DMEM HPL. Although not statistically significant, BI Media plus fibronectin coating provided the most adhesive surface (Fig. 1F). These cells grew up to p7 achieving 5.5 ± 2.2 population doublings and a cell number of 1.4 × 10^8^ ± 1.43 × 10^8^, they had positive vimentin and negative α smooth muscle actin (αSMA) staining suggesting that they maintain their fibroblast phenotype (Fig. 1G,H).

The incubation time for 10 µM MMC growth arrest of the buccal fibroblasts was investigated using xCELLigence to measure cell growth rate by electrical impedance. Two hours exposure to 10 μm MMC induced fibroblast cell stasis in the absence of significant decline in cell number and was used for feeder layer creation (Fig. 1I).

The final protocol for the fibroblasts was automated digestion followed by 3 days culture in Bi media on Fibronectin coated plates, then transferring to DMEM plus HPL for further media changes and passages.

### Epithelial cells

#### Buccal mucosal tissue was used for all epithelial cell experiments as a direct comparison to human tissue

**Figure 2:**
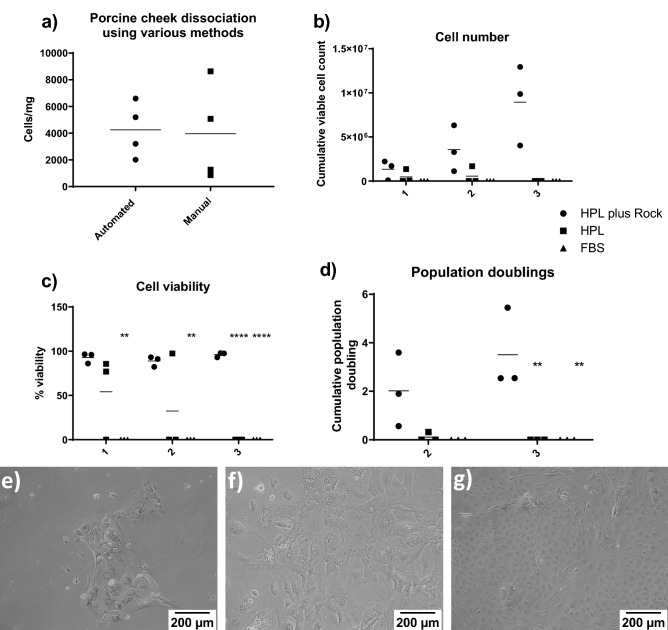
Epithelial cells digestion. The Porcine epithelial cell sheets were digested (**a**) either manually or automatically and the cell number counted (**a**). Data shows mean ± SD, *n* = 4. Comparison of epithelial cells automatically digested, grown on primary feeder layers in FBS, HPL or HPL Y-27632 medium. Cell number (**b**) viability **(c**) and population doublings (**d**) were recorded up to passage 3. Data shows mean ± SD; n = 3. *P ≤ 0.05, **P ≤ 0.01, ****P ≤ 0.0001 except **d**, which shows median + interquartile range. Cells grown in media containing FBS (**e**), HPL (**f**) and HPL plus Y-27632 (**g**). *n* = 4, scale bar shows 200 µm.

Digestion of the epithelial sheets in TrypLE was investigated. Buccal epithelial sheet was either, manually minced and incubated for 30 min at 37 °C, triturating every 10 min or placed into the enzyme cocktail and automatically dissociated using the Octodissociator program “37_TDK_1”. There was no significant difference between the cell numbers obtained by the two methods (Fig. 2A). For all further experiments the Octodissociator was used to standardise the protocol.

The composition of medium was investigated. We compared Epithelial medium containing 10% FBS, 5% HPL and 5% HPL plus 5 µM Y-27632. Cells grown in FBS-containing medium were flat, did not form the expected cobblestone pattern and did not survive to passage 1. HPL provided more viable cells, but after passage 2 these cells perished. The addition of Y-27632 improved the appearance of the colonies formed when viewed under a light microscope (Fig. 2E,F). There were significantly more cells grown in HPL + Y-27632 by passage 3, with a significantly higher viability of 96 ± 2.7%. These cells had undergone significantly more population doublings at 3.5 ± 1.7 by passage 3, with an average of 7.16 ± 4.3 days per population doubling (Fig. 2B–G). 5 µM Y-27632 was used in all further experiments.

We propose that autologous fibroblast feeders and epithelial cells can be obtained from the same biopsy. The removed epithelial cell sheet requires cryopreservation to allow development of an autologous feeder layer. The epithelial sheets were removed and cryopreserved directly in 4.5% HSA with 10%DMSO and 10% Y-27632. The epithelial cells were cryopreserved as a sheet to reduce operator error during digestion. Those frozen without Y-27632 did not survive. All cells from here have been cryopreserved prior to digestion.

### Comparison of conditions

**Figure 3:**
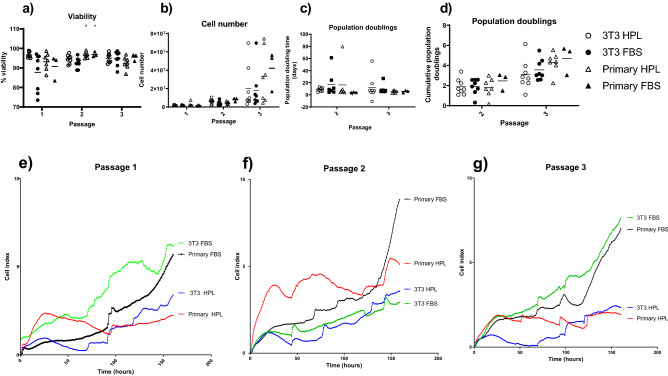
Comparison growth conditions. Porcine epithelial cells grown in the 4 conditions 3T3 FBS Y-27632, 3T3 HPL Y-27632, Primary FBS Y-27632, Primary HPL Y-27632 were compared. Viability (**a**), at passage 2 3T3 FBS is significantly reduced compared to those grown on primary feeder layers, cumulative cell number (**b**), population doubling time, data shows median plus interquartile range (**c**) and cumulative population doublings. Graphs show mean plus SD *P < 0.05, n = minimum of 3. xCELLigence growth at each passage 1 (**d**) passage 2 (**e**) Passage 3 (**f**). Wells were baselined to those containing feeder cells. Cell index is a measure of electrical impedance. Graphs show mean, Green shows 3T3 FBS, Blue shows 3T3 HPL, Black shows Primary FBS and Red shows Primary HPL. There is no significant difference between conditions at 160 h. n   =  3.

The epithelial cells created with this protocol were compared to those created by the industry standard protocol of 3T3 J2 feeder layers with FBS + Y-27632, as well as 3T3 J2 feeders with HPL + Y-27632 and primary feeder layers with FBS + Y-27632. There was no significant difference in cumulative cell number population doubling time and cumulative population doublings between the four methods, with 2 × 10^7^ ± 2.4 × 10^7^ 3T3 HPL, 1.8 × 10^7^ ± 2.2 × 10^7^ 3T3 FBS, 3.3 × 10^7^ ± 2.9 × 10^7^ primary HPL and 4.2 × 10^7^ ± 2.2 × 10^7^ Primary FBS cells achieved by passage 3. However there was a significant decrease in viability of cells grown on 3T3 J2 feeders with FBS medium at passage 2 (Fig. 3A–D). The comparable growth characteristics were confirmed when the epithelial cells were grown on the xCELLigence impedance plates, where we saw no significant difference in cell index (measure of cell density) at 160 h, although those grown in FBS-supplemented media achieved a higher cell index regardless of which feeder layer they were seeded on (Fig. 3E–G).

**Figure 4:**
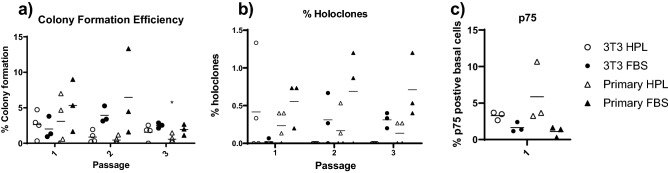
Stem cell populations. Stem cell analysis was carried out on porcine epithelial cells grown in the 4 conditions 3T3 FBS Y-27632, 3T3 HPL Y-27632, Primary FBS Y-27632, Primary HPL Y-27632,for Colony formation efficiency (CFE) assay all colonies were counted, by passage 3 those on primary HPL Y-27632 are significantly reduced compared to FBS 3T3 Y-27632 *P > 0.05 (**a**). Holoclones colonies larger than 10 mm with round edges were counted, there was no significant difference between the conditions (**b**). P75 was measure using Flow cytometry at passage 3. There was no significant difference between the conditions (**c**). Data shows mean plus SD, n = 3.

As the cells showed comparable growth patterns in each method, we extended the study to include analysis of epithelial stem cells. Colony formation efficiency was measured and this was significantly reduced by passage 3 for those cells grown on Primary feeders in HPL-supplemented media (Fig. 4A). However, analysis of the frequency of holoclones there was no significant difference between the groups suggesting that there was a reduction in transiently amplifying cells in cultures maintained on primary feeder cells but no loss of epithelial stem cells (Fig. 4B). This is supported by the retention of the cell subset within the buccal epithelial cells which expressed the stem cell marker p75. There was no significant difference in the percentage of p75+ cells between the four culture conditions (Fig. 4C).

**Figure 5:**
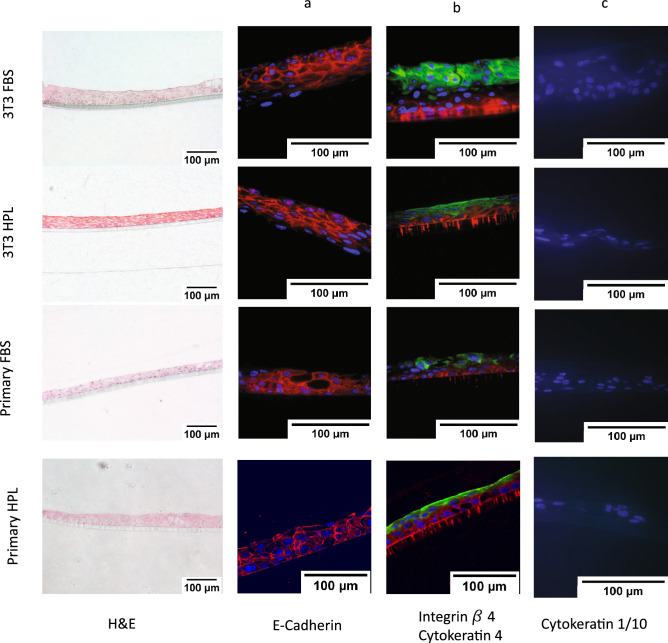
Immunofluorescent staining of the ALI cultures. Representative images for H &E, E-cadherin (red) (**a**) or integrin B4 (red) and cytokeratin 4 (green) (**b**) and Cytokeratin 1/10 (green) (**c**) for the 4 conditions 3T3 FBS Y-27632 (1st row), 3T3 HPL Y-27632 (2nd row), Primary FBS Y-27632 (3rd row), Primary HPL Y-27632 (4th row), Nuclei stained by DAPI (blue) Scale bar shows 100 µm, n = 3.

The cells were then grown in a non-contact co culture to investigate their ability to create a cohesive cell sheet. All conditions allowed the epithelial cells to form cell–cell junctions (Fig. 5a) and differentiate into multi-layered cell sheets (Fig. 5b), with HPL 3T3 4.3 ± 0.6, FBS 3T3 5 ± 1, Primary HPL 2.7 ± 0.6 and Primary FBS 3.3 ± 0.6 layers created, and were negative for the keratinised epithelial cytokeratin’s 1/10 (Fig. 5c).

### Human biopsies

**Figure 6:**
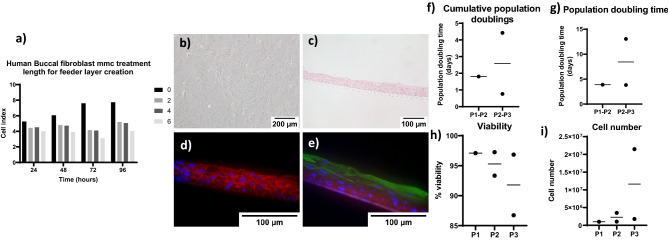
Human buccal cells. Fibroblasts underwent automated digestion and were plated onto fibronectin coated tissue culture plasticware for 3 days before media was changed to DMEM + HPL for further passages. Fibroblasts underwent MMC treatment for 0, 2, 4 and 6 h. 2 h stops cell proliferation without promoting apoptosis (**a**) n = 1. The epithelial cells underwent automated digestion and were plated onto a growth arrested human fibroblast feeder layer in HPL + Y-27632 media. At passage 3 they were seeded into a non-contact co-culture with human growth arrested fibroblast feeder layer. They underwent submerged culture for 3 days and were lifted to ALI for 14 days to allow differentiation. Phase image shows cells at p0 (**b**) H &E (**c**), E-cadherin staining (**d**) and cytokeratin 4 (green) found in intermediate and differentiated cells and Basal cell mark integrin β4 (red) (**e**) when differentiated at ALI. Population doublings (**f**), population doubling time (**g**), viability (**h**) and cell number (**i**), up to passage 3 were recorded, Scale bar shows 200 µm and 100 µm, n = 2.

The developed protocol was repeated with Human Buccal biopsies, fibroblasts cells were extracted, treated with MMC as above and, after seeding, progressed to form a confluent monolayer (Fig. 6A). The epithelial cells grown on the primary fibroblast feeder layer were able to form the distinctive cobblestone pattern (Fig. 6B) and were able to form adherens junctions and differentiate into a multi-layered cell sheet at ALI in a non-contact co-culture (Fig. 6C–E) with 4 ± 2.8 layers. The epithelial cells continue expanding up to passage 3 where 2.6 ± 2.6 population doublings (Fig. 6F) had occurred with a population doubling time of 8.4 ± 6.5 days (Fig. 6G), maintaining a viability of 91.8 ± 7.2 (Fig. 6H) with a total cell number of 1.2 × 10^7^ ± 1.34 × 10^7^ (Fig. 6I) from an interpolated starting cell number of 4.6 × 10^5^ ± 2.5 × 10^5^. The two samples both maintained p75 stem cells with 13.13 ± 1.7 and 6.4 ± 1.8 percent of basal cells. We estimate that this protocol applied to a human buccal tissue biopsy of no more than 1 cm^2^ would provide sufficient cells to manufacture a tissue engineered cell sheet of 17 cm^2^^30^.

## Discussion

Specifications for a regulatory compliant epithelial cell tissue engineered product for clinical use include expansion of cells to required number, retention of high quality, such as intercellular junction formation, and maintenance of differentiation capacity. Results from this study suggest that both epithelial and fibroblast cells can be obtained from a buccal biopsy in a GMP-compliant manner, which are not inferior to the current gold standard method yet avoid the need for a master cell bank tested to ICH Q5 standards. All products used within this protocol are available in GMP compliant versions or are risk assessed and approved for use by the European Medicines agency (EMA).

Obtaining both fibroblast and epithelial cell types from a single biopsy limits the effect on the patient. Oral biopsies are easy to obtain, under local anaesthetic with little morbidity or scarring^4^. The use of autologous cells for transplantation removes the need for immunosuppression^36^.

Removal of an epithelial sheet from the lamina propria by Neutral protease II (also known as Dispase II)^37,38^, has been used at various incubation times, temperatures^36,38–40^, and concentrations^36,37^ then either removed with forceps^41^ or scraped^42^. 4u of Neutral protease II for 16 h at 4 °C plus 10 min at room temperature allowed the whole sheet to be removed using forceps. Fibroblasts are obtained by collagenase digestion^30,43^ or outgrowth^44^. Digestion was chosen as it is quicker. Digestion of the epithelial cells is carried out in trypsin–EDTA solution^37,38^, TrypLE is an equivalent animal free alternative. The GentleMACS was used to standardise the homogenisation of the tissue during digestion. The initial fibroblast digestion was carried out using supraglottal tissue to increase the tissue samples available from a limited number of animals. The two tissues were compared with no significant difference in viable cell number or population doublings achieved. All further investigation using Buccal tissue.

FBS is a problematic component for many reasons, the potential risk of transmission of xenogenic antigens and pathogens, animal welfare issues as well as climate change issues associated with cattle, batch variation and limited volume^16^. HPL is a suitable replacement, it is created from expired platelets from apheresis or platelet rich plasma. Large pooled batches reduce batch variation^16^. It has been shown to aid corneal surface healing and epithelialization^45^.

Fibroblast growth was improved by the addition of HPL, although adhesion was reduced. The mechanisms for these observations are not fully understood, although fibronectin and vitronectin in FBS may contribute^46^. This lack of adhesion may be overcome by the use of a human fibronectin as a coating and a richer medium for the 1st 3 days to allow for attachment^47^. Fibroblast in a wound healing environment may undergo differentiation into myofibroblasts^48^. The cells here did not contain αSMA a marker for myofibroblasts, but did show the fibroblast marker vimentin suggesting that fibroblasts were obtained and maintained from this method.

Growth arrest was achieved after two hours of MMC exposure for both porcine and human fibroblasts. This is similar to observations for human oral fibroblast feeder layers treated between 2.5^49^ and 3 h^50^. After 5 days, the cells start to undergo apoptosis thereby reducing contamination risk to the final product.

The addition of the ROCK inhibitor Y-27632 provided cells with improved morphology and growth. Y-27632 has been used previously to aid the proliferation of epithelial cells^51^. Its suggested mode of action is reduction of differentiation, allowing the cells to retain stem cell-like characteristics both with^52^ and without 3T3 J2 feeder cells^53^ or to reduce anoikis^54,55^ and epithelial mesenchymal transition^56^ due to its action on the actin cytoskeleton^57^. Eye drops have been used to treat corneal damage^58^. It has also been shown to improve the cell viability of embryonic stem cells after cryopreservation. Buccal epithelial cells cryopreserved in HSA plus DMSO without Y-27632 were not viable. The addition of a cryopreservation step in a cell therapy is important, allowing the production to be timed in alignment with the patient treatment; reducing any additional waiting or further surgical procedures.

Impedance readings show the growth of adherent cells by the increase in impedance as more cells cover the surface of the electrodes. However epithelial cells form electrically tight barriers once their tight junctions have formed. The results in Fig. 3 suggest that these junctions are created earlier in the cells grown in FBS medium. As these are both undefined the exact reason for this is no yet known. However it appears that the junction formation catches up as the junctions are apparent in Fig. 5.

Although 3T3 J2 have been utilised for many years, there is little understanding on the exact reason for their superiority as even other murine embryonic fibroblast do not support epithelial culture in the same manner^59^. The 3T3 FBS model appears to provide the best cell sheet with a greater number of cell layers and definite differentiation, as expected for the current gold standard protocol, there was no detrimental effect seen in retaining stem cells between the conditions and all cells were able to form differentiated multi-layered cell sheets at ALI. Porcine buccal epithelial cells have been shown to have lower CFE capacity compared to human buccal epithelial cells, however they maintain their ability to form cell sheets^60^. P75 is a buccal epithelial stem cell marker^61^. It is these stem cells/holoclones which have been shown to promote the survival of a cell sheet in vivo^23^.

As there was no significant difference between the conditions, human buccal biopsies were digested and the cells tested with primary feeder layers and HPL medium. The human cells have a slower growth rate than the porcine, seen by the lower population doublings achieved 2.95 ± 2.6 vs 4.3 ± 1 by passage 3. Increased population doubling time of 8.4 ± 6 days (Human) vs 5.4 ± 2 days (Porcine) lead to a lower cumulative cell number being achieved by passage 3, 1.2 × 10^7^ ± 1.4 × 10^7^ (Human) vs 3.4 × 10^7^ ± 2.9 × 10^7^ (Porcine). The porcine cells are taken from 1 week old pigs, whereas the human tissues where taken from adults and it is established that the growth rate of cells decreases with age. However, despite this, the number of epithelial cells cultured would be sufficient to create a tissue engineered construct of 17 cm^2^^62^ for the reconstruction of the upper aerodigestive mucosa. Which is larger than the spilt skin grafts of 2 × 6 cm used for paediatric laryngotracheal stenosis^63^. All epithelial cells created were able to differentiate at ALI suggesting that they were good quality cells.

Porcine cells made a good model here as they gave comparable results to the human tissue and for the testing of the cell sheet within a pig which is a suitable animal for upper aerodigestive disorders^30^.

In conclusion, we have developed a prototype process for the creation of mucosal epithelial cells with sufficient quality and quantity to form the basis of a clinically useful product, without recourse to cells or reagents that would present regulatory issues or clinical risk.

## Conclusion

Both fibroblasts and squamous stratified epithelial cells can be obtained from a small, easily accessible, buccal biopsy and grown in entirely regulatory-compliant conditions. These cells are able form cell–cell junctions and maintain their stem cell niche and differentiation capacity. This prototype protocol forms a useful platform for the generation of clinically useful tissue-engineered mucosal replacements for unmet, and imperfectly met, surgical needs.

## Methods

### Cell sources

Buccal and supraglottal mucosal biopsies were taken from 7 day old piglets within 24 h of sacrifice. Human buccal pinch biopsies were obtained during routine ENT surgery after informed consent (in accordance with the Declaration of Helsinki for research involving human subjects). With ethical approval from RFL B-ERC part of the Royal Free London (RFL) NHS Foundation Trust Biobank (National Research Ethics Service, NRES, approved Research Tissue Bank) (16/WA/0289). Samples were processed within 2–6 h of harvest. Samples were obtained from a 72 and a 42 year old donor.

### Biopsy processing

Biopsies were digested by manual or automated means. For both protocols the biopsies were incubated overnight at 4 °C in 4U Neutral protease (30301.11 Amsbio, Abingdon, UK) 20 µl Antibiotic:Antimycotic (Anti-Anti), ciprofloxacin and gentamycin. The epithelial sheet was removed using forceps.

### Fibroblast cell acquisition

The lamina propria was either minced and placed into 0.3U/ml collagenase NB4 (17454.01, Amsbio, Abingdon, UK) with 229U/ml Pulmozyme (dornase alpha, Genentech, San Francisco, USA) and 1% Anti:Anti, 250 µg ciprofloxacin and 25 µg gentamycin and incubated for 3 h at 37 °C in 5% CO_2_ in air, or placed into the enzyme cocktail for 3 h at 37 °C, and dissociated using GentleMacs Octodissociator (Miltenyi Biotec, Woking, UK) program h_Skin_1. The cells were washed with low glucose Dulbecco’s Modified Eagle Medium (DMEM) and passed through a 100 µM cell strainer, and plated into a T25 tissue culture flask (Corning, Thermo Fisher, UK Ltd). DMEM plus foetal bovine serum (FBS) plus Non-Essential amino acids (NEAA).

### Epithelial cell acquisition

The epithelial sheet was either minced then placed into TrypLE Express (Gibco, Life Technologies, Paisley, UK), 229 U/ml Pulmozyme, 1% Anti:Anti, 250 µg ciprofloxacin and 25 µg gentamycin, then incubated for 30 min at 37 °C, triturating every 10 min, or placed into the enzyme cocktail and dissociated using the octodissociator program 37_TDK_1. The cells were washed with DMEM and passed through a 100 µM cell strainer, then plated in Epithelial media containing 330 ml (DMEM), 110 ml Ham's F12 Nutrient Mixture (F-12, Sigma Aldrich Ltd, Poole, UK), 10% FBS or 5% Stemulate pooled human platelet lysate (HPL) (Cook Regentec, Indianapolis, USA), 0.4 µg/ml hydrocortisone (R&D systems Inc, Minnesota, USA), 10 µg/ml human recombinant epidermal growth factor (Bio-Techne Ltd, Abingdon, UK), 5 µg/ml insulin (Actrapid, Novo Nordisk, Bagsvaerd, Denmark), 250 µg isoproterenol (Calbiochem, Merck Millipore, Massachusetts, USA), 1% Anti:Anti, 250 µg ciprofloxacin and 25 µg gentamycin, which was modified during the investigation.

### Cell culture

At 70–80% confluency the epithelial cells were washed with PBS. The feeder layer was removed using 5 ml 0.2% ethylenediaminetetraacetic acid (EDTA). This was removed and 5 ml of TrypLE added. Digested cells were diluted with 10 ml of epithelial media. Cells were centrifuged at 300×*g* for 5 min (Allegra X-15R, Beckman Coulter, UK) before being re-suspended in Eps-Media containing 5 µM ROCK inhibitor Y-27632 (Cell guidance systems, Babraham, UK). At 70–80% confluency the fibroblast cells were washed with PBS and 5 ml TrypLE was added and the cells incubated for 5 min at 37 °C. Digested cells were diluted with 10 ml DMEM 10% FBS, 1% Anti:Anti, 250 µg ciprofloxacin and 25 µg gentamycin. 3T3 J2 cell line (Kerafast, Boston, USA) at 60–80% confluency the cells were washed with PBS (Sigma Aldrich Ltd, Poole, UK) and 5 ml TrypLE was added and the cells incubated for 5 min at 37 °C. Cells were seeded at 3.5 × 10^3^ cells/cm^2^ in DMEM, 10% bovine calf serum iron supplemented (GE healthcare, Amersham, UK). All cell lines were continuously cultured at 37 °C in 5% CO_2_ in air.

### Creation of feeder layers

The primary and 3T3 J2 fibroblasts were treated with 10 µM Mitomycin (MMC) (Kyowa Kirin Ltd, Galasheils, UK) for two hours before washing with PBS and seeding at 1 × 10^4^ cells/cm^2^ (primary) or 2 × 10^4^ cells/cm^2^ (3t3).

### Cell adhesion

A black walled 96-well plate (Costar, Corning Inc, New York, USA) was set up with 7 conditions, coatings of BSA as a negative control, recombinant human Fibronectin (05-752, MSC Attachment Solution, Biological Industries, Beit-Haemek, Israel) with DMEM plus 5% HPL and MSC NutriStem XF Media plus MSC NurtiStem XF Supplement Mix (BI media) (05-200, 05-201 Biological Industries, Beit-Haemek, Israel) plus 5% HPL and DMEM plus 10% FBS, DMEM plus 5% HPL, BI media Plus 5% HPL with no coatings. After digestion, fibroblasts were incubated with Calcein AM for 30 min. The Calcein AM stained cells (3 × 10^4^) were added to a black walled 96-well plate and incubated for 3 h before being washed 3 times with PBS. A standard curve of Calcein stained cells of known concentration was set up, with fluorescence read at 485 excitation, 535 emission on Victor × 3 multimode plate reader (Perkin Elmer, Massachusetts, USA). The cell number was extrapolated from a standard curve using GraphPad Prism. Data represents 3 independent cell lines in triplicate.

### xCELLigence growth assay

Fibroblasts were treated with 10 µM MMC for 0, 2, 4, 6, 8 and 18 h, washed three times with PBS before being passaged. Then 4800 cells per well seeded onto Glass E-plates (Agilent, Santa Clara, USA). The plate was placed into the xCELLigence plate reader (Agilent) and impedance read at 1 h intervals for 120 h with 1 media change. Date represents 3 independent cell lines in duplicate.

### xCELLigence Epithelial cell growth

Feeder layers were seeded onto Glass E-plates. After 2–24 h the epithelial cells were seeded on top of half of the feeder layers, the plate was placed into the xCELLigence plate reader, impedance was read at 1 h intervals for 160 h with 2 media changes. The epithelial cell index readings were baselined against the feeder layers (RTCA software pro) to obtain information on only epithelial cells. The cell index was then plotted in GraphPad Prism 8.4. Data represents 3 independent cell lines in triplicate.

### P0 calculation

Seeding = harvest / [e ^ (specific growth rate X culture time)].

### Colony formation efficiency (CFE)

Triplicate feeder layers were set up in a 6 well plate and 500 epithelial cells added, these were incubated for 14 days at 37 °C, 5% CO_2_. All colonies or holoclones, colonies greater than 10 mm diameter with smooth edges, were counted and CFE calculated ((colony number/seeded cell number) × 100). Data represents 3 independent cell lines in triplicate.

### P75 stem cell analysis

Epithelial Cells from passage 3 were washed in flow buffer (PBS, 2% FBS), and incubated with p75 (Mab 5386x, Merck, Watford, UK) and Integrin β 6 (CD49f) (BD555736, BD, Wokingham, UK) for 15 min at 4 °C and run on NovoCyte flow cytometer (Agilent). Data from 1 × 10^5^ CD49f. positive events were analysed using NovoExpress flow analysis software (Agilent). Data represents 3 independent cell lines in triplicate.

### Creation of Non-contact co-cultures

2 × 10^4^ feeder cells were seeded into the base of the well. 1.2 × 10^5^ epithelial cells were seeded onto the top of a Transwell cell culture inserts, 0.4 µm pore size. These were submerged in Eps-media for 3 days, then brought to ALI for a further 14 days, media changed three times a week. Data represents 3 independent cell lines in triplicate.

### Immunofluorescent staining

The Transwell membranes and fibroblasts grown on sterile coverslips were fixed in 4% paraformaldyde, the Transwells were paraffin embedded, and sectioned to 4 µM. They were either, stained with H&E and examined under light microscope (Nikon eclipse Ti-E) or immunofluorescently (IF) stained. For IF staining the sections were deparaffinised using xylene and rehydrated. They underwent heat induced antigen retrieval in boiling Tris-EDTA-Tween buffer (10 mM Tris base, Sigma Aldrich Ltd, Poole, UK), 1 mM EDTA solution, 0.05% Tween 20, pH 9, for Cytokeratin 4 (GTX11215, GeneTex, Irvine, USA), Integrin β4 (sc-9090, Santa Cruz Biotechnology Inc, Dallas, USA), and Cytokeratin 1/10 (sc-53251, Santa Cruz), or Citrate buffer (1.92 g anhydrous citric acid, 0.5% tween 20, pH6) for E-Cadherin (ab15148, Abcam, Cambridge, UK) for 20 min. The fibroblasts were permeabilised for 15 min in 0.1% triton x-100 in PBS. Then both were blocked for 1 h in 1% BSA, 0.1% Tween 20 in PBS and incubated with primary antibodies (vimentin, ab8069, Abcam and αSMA 1A4/asm-1 Bio-Techne Ltd, Abingon, UK), overnight at 4 °C. Then incubated with Secondary antibodies anti-mouse Alexa 488 and anti-rabbit Alexa 633 (Life Technologies) for one hour in the dark at room temperature. Cell nuclei were visualised using DAPI (diamindino-2-pheylindole) and mounted using Prolong Gold antifade reagent. These were visualised using a fluorescent light microscope (Olympus Ix70) equipped with a red filter cube (excitation 600, emission 650 nm) a UV filter cube (excitation 350 nm, emission 450 nm) and a green filter cube (excitation 450 nm, emission 550 nm) or a laser scanning confocal Microscope system (Nikon C2 with LU-N4 laser unit) imaging of DAPI (405 nm) Alexa 488 (488 nm) and Alexa 633 (640 nm) and displayed with pseudo-colouring. The images were merged using Image J. Data for Fibroblast represents 3 independent cell lines in triplicate. Data for porcine samples represents 3 independent cell lines with a single Transwell. Data for Human samples represents 2 independent cell lines in triplicate.

### Statistical analysis

Data were tested for normality using the Shapiro–Wilk test. Parametric data are shown as means ± standard deviation (SD). The difference between two groups was tested for significance using paired T test and the differences between > 2 groups was tested for significance using two way ANOVA with multiple comparisons. Non-parametric data are shown as median ± interquartile range, and the difference between groups was determined by Kruskal–Wallis with Dunn’s correction for multiple comparisons. All data were analysed using Prism (GraphPad Software, San Diego, USA). P < 0.05 were considered statistically significant.

## Data Availability

Data are available in UCL repository https://doi.org/10.5522/04/13862309.
